# Foreign Body Granuloma After Embolization of Internal Iliac Artery Aneurysm Using N-Butyl-2-Cyanoacrylate: A Case Report

**DOI:** 10.7759/cureus.60187

**Published:** 2024-05-13

**Authors:** Masaya Fumimoto, Shigeshi Kohno, Shojiro Oka, Yuko Someya, Reiichi Ishikura, Ken Nakamura, Daisuke Yamashita, Hiroyuki Ueda, Kumiko Ando

**Affiliations:** 1 Diagnostic Radiology, Kobe City Medical Center General Hospital, Kobe, JPN; 2 Cardiovascular Surgery, Kobe City Medical Center General Hospital, Kobe, JPN; 3 Pathology, Kobe City Medical Center General Hospital, Kobe, JPN; 4 Radiology, Kokura Memorial Hospital, Kokura, JPN

**Keywords:** interventional radiology, embolization, internal iliac artery aneurysm, lipiodol, n-butyl-2-cyanoacrylate, foreign body granuloma

## Abstract

Foreign body granulomas following endovascular treatment are rare complications and are mostly reported in the brain or cutaneous vascular tissues. To the best of our knowledge, no study to date has reported on foreign body granulomas in the abdomen after injection of N-butyl-2-cyanoacrylate (NBCA)-lipiodol mixture into the abdominal arteries. This study reports a case of foreign body granuloma that appeared 12 months after the embolization of a right internal iliac artery aneurysm using an NBCA-lipiodol mixture, which posed challenges in differentiation from malignant tumors. We present a 77-year-old man who underwent embolization of a right internal iliac artery aneurysm and open surgical repair of an abdominal aortic aneurysm. A contrast-enhanced CT performed 12 months postoperatively revealed a right-sided retroperitoneal mass surrounding the iliopsoas muscle. The mass contained multiple, small, hyperdense areas, suggesting the migration of the NBCA-lipiodol mixture casts from the embolized right internal iliac artery aneurysm. The differential diagnosis included foreign body granuloma, lymphoma, and sarcoma. A biopsy of the lesion revealed a granuloma with various stages of inflammation, no hemosiderin deposition, multinucleated giant cells, and foam cells containing fat, and was diagnosed with a foreign body granuloma. Special staining for microorganisms revealed no findings suggestive of infection. Because the patient was asymptomatic, no treatment was administered. Contrast-enhanced CT at 24 months postoperatively showed shrinkage of the mass, with no change in size noted at 48 months postoperatively. This report highlights a foreign body granuloma that mimicked malignant tumors. Extravascular migration of the NBCA-lipiodol mixture casts likely contributed to granuloma formation. Radiologists should consider foreign body granulomas after embolization using NBCA into the abdominal arteries.

## Introduction

Foreign body granulomas have recently emerged as a rare complication of endovascular treatment [1-13], mostly reported in the brain or cutaneous vascular tissues. These granulomas pose diagnostic challenges, particularly when presenting as mass formation on imaging, often mimicking neoplasms. Despite the increasing recognition of this entity, to the best of our knowledge, no studies have reported on foreign body granulomas after the injection of a mixture of N-butyl-2-cyanoacrylate (NBCA) and lipiodol (Guerbet, Aulnay-Sous-Bois, France) into the abdominal arteries. This study reports a case of foreign body granuloma after embolization of the internal iliac artery using an NBCA-lipiodol mixture, which presented diagnostic difficulty in differentiating from malignant tumors.

This article was previously presented as a meeting abstract at the Interventional Radiology Case Club 2023 on Nov 18, 2023, and posted to the Research Square preprint server on Mar 13, 2024.

## Case presentation

**Figure 1:**
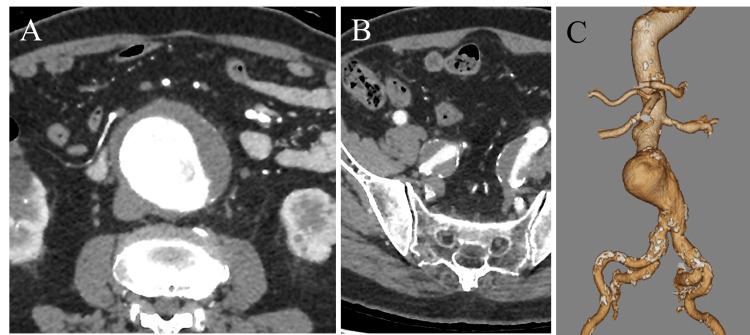
Preoperative images A contrast-enhanced CT showing an AAA measuring 64 mm, a right internal iliac artery aneurysm measuring 28 mm, and a left internal iliac artery aneurysm measuring 22 mm. (A,B) Arterial phase. (C) Volume-rendering image.

A 77-year-old man presented with an incidentally detected abdominal aortic aneurysm (AAA). His medical history included hypertension, chronic kidney disease, and cerebral hemorrhage. A contrast-enhanced CT revealed an AAA measuring 64 mm, a right internal iliac artery aneurysm measuring 28 mm, and a left internal iliac artery aneurysm measuring 22 mm (Figure 1). An embolization of the right internal iliac artery aneurysm was performed before open surgical repair of the AAA using a Y-graft.

**Figure 2:**
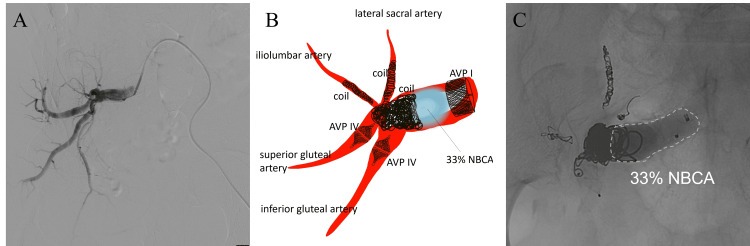
Intraoperative angiographic images and schema (A) Initial angiography of the right internal iliac artery. (B) A schematic drawing of the embolization of the right internal iliac artery aneurysm. The iliolumbar artery and the lateral sacral artery are embolized with coils. The superior gluteal artery and inferior gluteal artery are then embolized with vascular plugs. Next, the aneurysm is embolized with coils and NBCA mixed with lipiodol at a ratio of 1:2. Finally, the proximal part of the internal iliac artery is embolized with a vascular plug. (C) A post-embolization fluoroscopic image shows successful embolization of the aneurysm. The migration of the NBCA-lipiodol mixture casts is not shown.

An endovascular intervention was performed through retrograde access via the left common femoral artery. A 5-Fr diagnostic catheter (Cobra type; Medikit, Tokyo, Japan) was inserted through a 6-Fr sheath into the right internal iliac artery. A right internal iliac arteriography revealed an aneurysm and its distal branches (Figure 2). A 1.7/2.4-Fr microcatheter (Excelsior SL10; Stryker Neurovascular, Fremont, CA) and 5-Fr diagnostic catheter were advanced into each distal branch, which were embolized with coils (Target™; Stryker Neurovascular, Fremont) and vascular plugs (Amplatzer Vascular Plug IV; Abbott Vascular, Redwood City, CA). Subsequently, the right internal iliac artery aneurysm was embolized using coils and NBCA that was mixed with lipiodol at a ratio of 1:2. Finally, a vascular plug (Amplatzer Vascular Plug I; Abbott Vascular, Redwood City, CA) was added to the proximal right internal iliac artery. A post-embolization angiogram confirmed successful embolization of the aneurysm. The operative time was approximately two hours, without any significant complications. Subsequently, the patient underwent open surgical repair of the AAA seven days after embolization.

**Figure 3:**
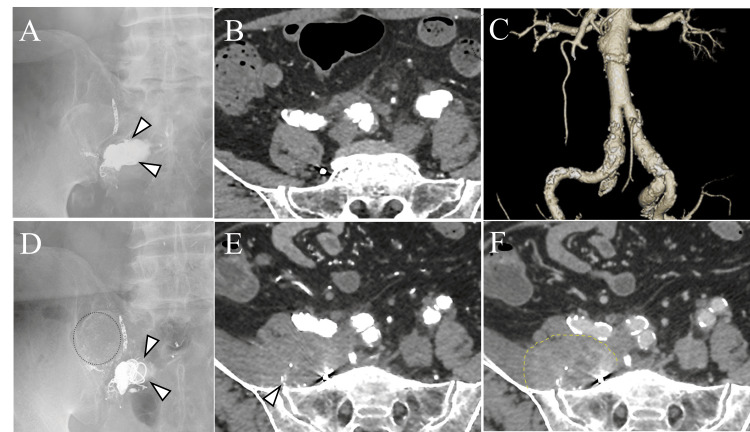
Postoperative images (A-C) An abdominal radiograph and contrast-enhanced CT obtained shortly after the embolization of the right internal iliac artery aneurysm and open AAA repair showed no migration of NBCA mixed with lipiodol (arrowheads). The shape of the coils and blood vessels is unchanged, and the AAA is repaired. (B) Arterial phase. (C) Volume–rendering image. (D) An abdominal radiograph obtained five months postoperatively shows the migration of the NBCA-lipiodol mixture cast around the coils (dotted line, arrowheads). (E, F) Abdominal contrast-enhanced CT obtained at 12 months postoperatively shows the appearance of a 70-mm irregular mass in the right retroperitoneum surrounding the iliopsoas muscle (dotted line). The lesion contains multiple, small, hyperdense areas suggested to be extravascular migration of NBCA-lipiodol mixture casts (arrowhead). The lesion had mild enhancement in the venous phase. (E) Arterial phase. (F) Venous phase.

Postoperative contrast-enhanced CT and radiography confirmed that the right internal iliac artery was occluded with no mass lesions around the embolized artery (Figures 3A-3B) and the AAA was repaired (Figure 3C). The patient was discharged 10 days postoperatively without any complications. Radiographs obtained at 5 months postoperatively showed migration of the NBCA-lipiodol mixture casts (Figure 3D). Follow-up contrast-enhanced CT at 12 months postoperatively revealed a 70-mm irregular mass in the right retroperitoneum surrounding the iliopsoas muscle (Figure 3E). The mass had mild enhancement in the venous phase (Figure 3F), and its border with the muscle was indistinct. Multiple small, hyperattenuated areas were observed within the mass, suggesting migration of the casts from the embolized right internal iliac artery aneurysm. A CT-guided biopsy was performed to investigate suspected foreign body granulomas or retroperitoneal malignant tumors such as sarcoma or lymphoma. The acquisition of specimens was repeated six times using a semiautomatic 18-G spring-loaded cutting needle, and a sufficient sample was obtained for pathologic evaluation.

**Figure 4:**
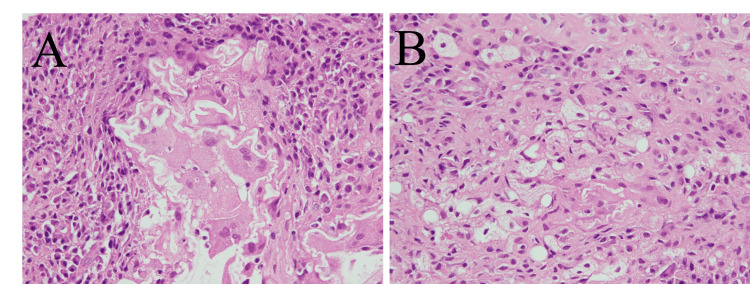
Histological examination (A,B) A histological examination (hematoxylin–eosin stain, x40) reveals multinucleated giant cells and foam cells containing fat in line with foreign body granuloma.

The specimen showed various stages of inflammation, multinucleated giant cells, and foam cells containing fat with no hemosiderin deposition, and was diagnosed with a foreign body granuloma (Figures 4A-4B). A special staining for microorganisms revealed no findings suggestive of infection. As the patient was asymptomatic, no treatment was administered. A contrast-enhanced CT at 24 months postoperatively showed shrinkage of the mass, with no further changes in the mass size observed at 48 months postoperatively.

## Discussion

Foreign body granuloma has been reported as a rare complication of endovascular treatment, often associated with hydrophilic polymers used as coatings on neuro-endovascular intervention devices that can cause granulomatous inflammation [1,2]. Additionally, foreign body granulomas because of embolic materials such as NBCA [3-5], onyx [6,7], and gelatin sponges [8] have been reported previously. Recent studies have reported delayed hypersensitivity reactions and granuloma formation after injection of NBCA into peripheral vessels [4] and esophageal varices [5]. 

In our case, a mass with scattered multiple small hyperdense areas, suggesting the migration of the NBCA-lipiodol mixture casts, enlarged around an embolized internal iliac artery aneurysm using NBCA at 12 months post-endovascular treatment. This mass posed challenges in differentiation from retroperitoneal malignancies. The hyperdense areas within the lesions on CT might help differentiate the lesion from other retroperitoneal tumors. A biopsy of this mass revealed foam cells containing fat with several multinucleated giant cells and inflammation cells, which are characteristic histopathological features of foreign body granulomas. The biopsy site in our case does not normally contain abundant fat, which suggests that the fat observed in foam cells most likely originated from lipiodol. The NBCA and lipiodol mixture was used as standard embolization procedure in our case, but no traces of NBCA could be directly identified in the specimen. In previous reports of foreign body granulomas after NBCA embolization [4,5], they were also not able to identify NBCA as a foreign body in the histologic sections. Furthermore, Calvo et al. [14] have reported that standard staining techniques do not provide the necessary differentiation between NBCA and lipiodol and have used the europium fluorescence visualization process to quantify NBCA within treated tissues [14]. However, the limited volume of the biopsy specimen could be a limitation, and we interpreted this consistent finding as indicative of granulomatous inflammation because of oil (lipiodol)-contained embolic material (i.e., NBCA).

Foreign body granuloma formation has been suggested to be associated with type IV allergies [2]. Previous cases involving NBCA treatment [4,5] have documented complete extrusion of casts from the target vessels, and the formation of granulomas was observed following NBCA treatment. In the present case, a mass also appeared with cast migration from the embolized right iliac artery aneurysm, which suggests that an allergic reaction to delayed extravascular extruded cast may have caused the granuloma formation. Although the mechanism of delayed extravascular extrusion remains unclear, cast extrusion has been observed in both arteries and veins after NBCA injections in animal models [9]. Embolization of the right internal iliac artery aneurysm may have caused inflammation and ischemia of the aneurysm wall, resulting in extrusion of the NBCA-lipiodol mixture casts. Parsi et al. [10] recommended against the use of cyanoacrylate adhesive closure in patients with uncontrolled inflammatory, autoimmune, or granulomatous disorders such as sarcoidosis. However, the patient in this report had no history of such disorders.

In this case, other potential exogenous materials include hydrophilic polymers and coils. First, hydrophilic polymers used in devices may cause foreign body embolisms, and foreign bodies may induce delayed granulomatous responses [1,2]. As our patient also underwent endovascular intervention using a hydrophilic-coated device, foreign body reaction to the hydrophilic polymer may have caused the foreign body granuloma. Although the histopathological examination of the specimens did not reveal polymer components, the evaluation was limited because of the number of biopsy specimens. Excess friction between devices and prolonged procedure times are factors resulting in the separation of device coatings [1,2]. In this patient, no difficulty was noted in accessing the right internal iliac artery aneurysm, and the procedure was completed without any complications. Therefore, the hydrophilic polymers were most likely not the cause of granuloma formation. Second, we also used coils in the treatment. An abdominal radiograph at five months postoperatively showed loosening of the coils, but the coils were located at the margins of the mass; thus, coils were unlikely to be involved in the formation of the granuloma. To the best of our knowledge, no reports of granuloma formation after using only coils are available.

Foreign body granulomas after endovascular treatment may cause various symptoms depending on the site of origin. As our patient had no symptoms and the lesion reduced in size during follow-up, no treatment was administered. In symptomatic patients, steroid treatments have shown efficacy against foreign body granulomas in the brain after infection is ruled out [11]. An optimal standard treatment for foreign body granulomas, including the dosage, has not yet been established. Athavale et al. [12] reported that a longer steroid taper should be considered in patients with type IV sensitivity or severe symptoms. Surgical removal of the lesion may be a therapeutic option in patients with severe symptoms [6]. However, several case reports indicate surgical treatment because of the misdiagnosis of a foreign-body granuloma as a tumor [5,13]. Radiologists must consider foreign body granulomas after endovascular treatment of the abdominal arteries.

## Conclusions

This report study presents a case of foreign body granuloma formed after the embolization of a right internal iliac artery aneurysm. As a histopathological examination of the biopsy specimen revealed foreign body granuloma containing fat, the extravascular migration of the NBCA-lipiodol mixture casts may have caused the granuloma formation. These granulomas can be difficult to differentiate from malignant tumors; however, migration of casts outside the target vessels on imaging may help make a diagnosis. Foreign body granulomas should be included in the differential diagnosis of mass lesions observed after embolization using NBCA into the abdominal arteries.
